# Need for Invasive Meningococcal Disease Prevention Through Vaccination for Young Children in the Americas

**DOI:** 10.3390/vaccines13090974

**Published:** 2025-09-13

**Authors:** Gaurav Mathur, Joseph B. Domachowske, Maria Gabriela Graña, Reena Ladak, Joanne M. Langley, Oluwatosin Olaiya, Alysa Pompeo, Laura Taddei, Rodolfo Villena

**Affiliations:** 1 GSK, Rockville, MD 20850, USA; 2 Department of Pediatrics, SUNY Upstate Medical University, Syracuse, NY 13210, USA; domachoj@upstate.edu; 3 GSK, Recoleta 8431633, Chile; maria.g.grana@gsk.com; 4 GSK, Singapore 139234, Singapore; reena.x.ladak@gsk.com; 5 Canadian Center for Vaccinology, Departments of Pediatrics and Community Health and Epidemiology, Dalhousie University, IWK Health and Nova Scotia Health, Halifax, NS B3K 6R8, Canada; joanne.langley@dal.ca; 6 GSK, Philadelphia, PA 19104, USA; tosin.o.olaiya@gsk.com; 7 GSK, Toronto, ON L5R 4B2, Canada; alysa.a.pompeo@gsk.com; 8 GSK, 53100 Siena, Italy; laura.x.taddei@gsk.com; 9 Hospital Dr. Exequiel González Cortés, Santiago 8900000, Chile; rvillena@uchile.cl; 10 Faculty of Medicine, Universidad de Chile, Santiago 1058, Chile

**Keywords:** invasive meningococcal disease, serogroup B, infants, vaccination

## Abstract

**Background:** Invasive meningococcal disease (IMD) is an uncommon but potentially life-threatening condition, resulting in life-long sequelae or death in up to 20% of cases. Most IMD cases are caused by Neisseria meningitidis serogroups (Men) A, B, C, W, X, and Y. The highest IMD incidence is among children < 5 years of age (YOA). We reviewed IMD epidemiology data and existing national immunization programs (NIP) in the Americas and identify unmet needs to decrease IMD burden in young children. **Methods:** Using national surveillance data and published literature from 2006 to 2024, we evaluated the IMD burden and national vaccination strategies for children < 5 YOA in the Americas, focusing on Canada, the United States, Brazil, Chile, Argentina. **Results:** The highest IMD incidence was among infants, followed by children 1–4 YOA, with MenB infections predominating in both age groups. Chile has both MenACWY (2014) and MenB (2023) infant vaccination in its NIP. Argentina and Brazil’s NIPs include MenACWY (2017) and MenC (2010) vaccinations for infants, respectively. In Canada, MenC (2002) vaccination is recommended at 1 YOA (replaced by MenACWY in 2024 in Manitoba); MenB vaccination is selectively recommended. In each country, the incidence of IMD caused by vaccine-preventable serogroups decreased following the introduction of the respective meningococcal vaccination in the NIP. **Conclusions:** Comprehensive meningococcal vaccination programs in the Americas have the potential to reduce the IMD burden in children < 5 YOA. National recommendations and NIPs could reduce IMD burden by offering equitable access to protection against IMD, aligning with the WHO roadmap to defeat meningitis by 2030.

## 1. Introduction

Invasive meningococcal disease (IMD) is an uncommon but life-threatening infection caused by the bacterium *Neisseria meningitidis*. The typical clinical presentation includes meningitis with or without sepsis, which leads to severe, life-long sequelae in up to 20% of survivors [1], with serious impact on the quality of life [2]. Even with appropriate and aggressive treatment that includes the administration of systemic antibiotics, case-fatality rates (CFRs) range between 4% and a staggering 20% [3]. The highest incidence of IMD and one of the highest CFRs occur among children < 5 years of age [4]. A second peak in IMD incidence is observed during adolescence [4,5] and a third peak in adults ≥ 65 years of age [6].

The epidemiology of serogroup-specific IMD varies widely and unpredictably over time and across geographic regions [7]. Globally, most IMD cases are caused by meningococcal serogroups A (MenA), B (MenB), C (MenC), W (MenW), X (MenX), and Y (MenY). In recent decades, MenB-IMD has become predominant, especially across Europe, North and South America, and Australia [7]. In Europe, the burden of IMD prompted the introduction of meningococcal vaccination in national immunization programs (NIPs) for young children and adolescents, with several countries implementing routine vaccination against serogroups A, B, C, W, and/or Y [8]. The COVID-19 pandemic was associated with decreased incidence of many communicable diseases, including IMD; however, since 2022, with the lifting of public health measures an increase in the number of IMD cases approaching pre-pandemic levels has been observed [9].

**Figure 1:**
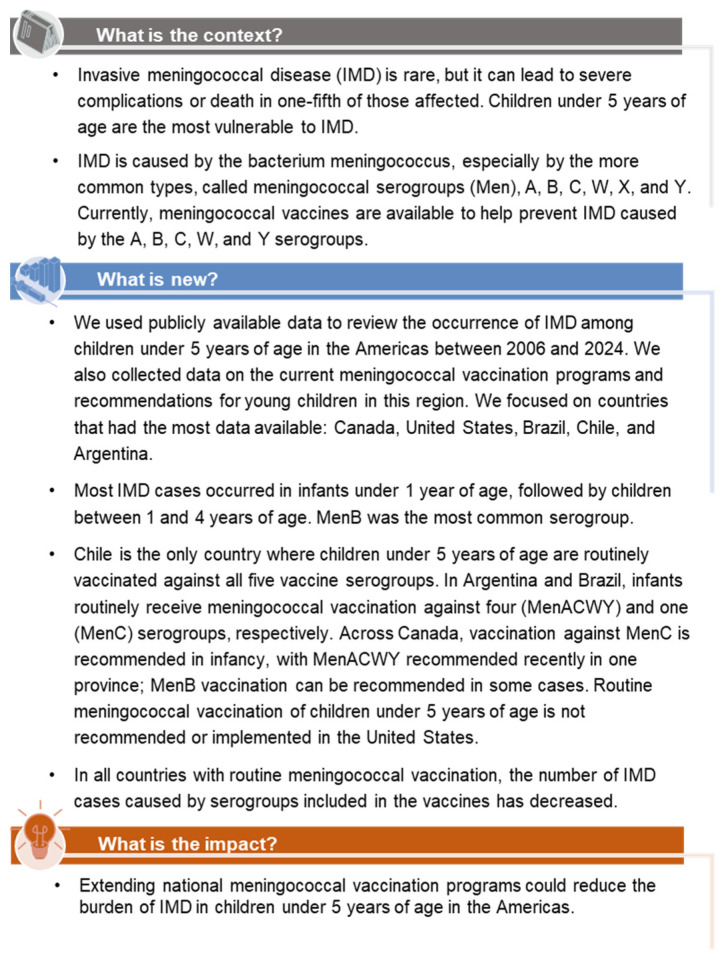
Plain language summary.

As the highest burden of IMD occurs among children < 5 years of age, we aimed to evaluate the regional IMD burden and vaccination strategies targeting infants and children 1–4 years of age in the Americas. An overview of our findings for non-expert readers is provided in the plain language summary (Figure 1).

## 2. Materials and Methods

We performed an online search (using the Google search engine) for national surveillance data on IMD epidemiology among children < 5 years of age and national/regional vaccination strategies, recommendations, and/or programs in this age group. We searched for information from Health Ministries in all countries in the Americas. When no other source of data was available for Latin American countries, we included the Regional System for Vaccines (SIREVA) II network surveillance data from 2006, i.e., the earliest year in which surveillance reports were available. In addition, on 5 December 2024, we performed a literature search in PubMed, using the string (meningococcal disease [Title/Abstract]) AND (((infants [Title/Abstract]) OR (children under 5 years of age [Title/Abstract])) OR (children 2–4 years of age [Title/Abstract])) and the name of each country for which recent IMD epidemiology data were available, as identified in the online search.

## 3. IMD Epidemiology and Implemented Vaccination Strategies

We found recent publicly available IMD epidemiologic data for Canada, the United States (US), Brazil, Chile, Argentina, Cuba, Costa Rica, Honduras, Panama, Colombia, and Uruguay. We focused the review on the countries for which the data were consistently reported over at least 5 consecutive years during the last decade: Canada, the US, Brazil, Chile, and Argentina. To provide a more comprehensive view of the IMD epidemiology in the Americas, we have also summarized available data for six other countries (Colombia, Uruguay, Panama, Honduras, Cuba and Costa Rica). Of note, the available surveillance data were derived from different sources and starting from different dates, even in the five countries with consistent reporting. IMD epidemiology and meningococcal vaccination programs from the remaining countries in the Americas region are not presented here, due to the absence of national surveillance data.

The PubMed search yielded 153 query hits, of which 27 were duplicates (identified in searches from more than one country). Of the 126 publications retained, only six [10,11,12,13,14,15] contained relevant data on IMD epidemiology or vaccination programs for the five main countries and were included in the review. Publications focusing on cost-effectiveness aspects of meningococcal vaccination were out-of-scope for this review and were therefore excluded.

### 3.1. Canada

Since 1924, IMD has been a notifiable disease in Canada at a national level, reported initially through the Canadian Notifiable Diseases Surveillance System, and then through the Enhanced Invasive Meningococcal Disease Surveillance System (eIMDSS; since 1992) [11,13,16]. eIMDSS is part of the Public Health Agency of Canada’s national surveillance system.

Routine immunization against MenC was introduced in 2002 [17], and vaccination is recommended as a single dose administered at 12 months of age [18]. By 2007, the program was implemented in all Canadian jurisdictions. A steep decrease of 43% was observed in the mean incidence of MenC disease in the overall population during 2000–2005 (during which time all but one jurisdiction had implemented routine MenC immunization) as compared to the pre-vaccination era (1995–2001) [17].

**Figure 2:**
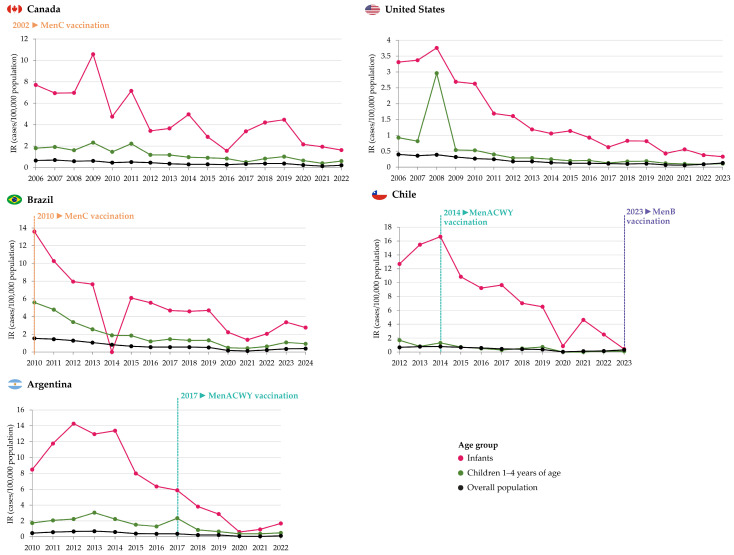
Incidence of IMD in Canada, the United States, Brazil, Chile, and Argentina, by year, overall and by age group. IMD, invasive meningococcal disease; IR, incidence rate. Note: ► indicates the timing of the introduction of meningococcal vaccination in the national immunization program or publicly funded vaccination for children < 5 years of age. In Brazil, the IR in infants for 2014 was reported as 0.0 in [19], but 202 cases were reported. Incidence data for Argentina are estimates obtained from the digitalization of Figures 1 and 2 in [20], using https://www.graphreader.com/ (accessed on 4 March 2025).

During 2006–2011, the overall mean age-adjusted annual incidence rate of IMD due to all serogroups was 0.60/100,000 population. The age-specific incidence was significantly higher among infants (7.35/100,000 population) and children aged 1–4 years (1.89/100,000 population) than the incidence among other age groups (Figure 2).

**Figure 3:**
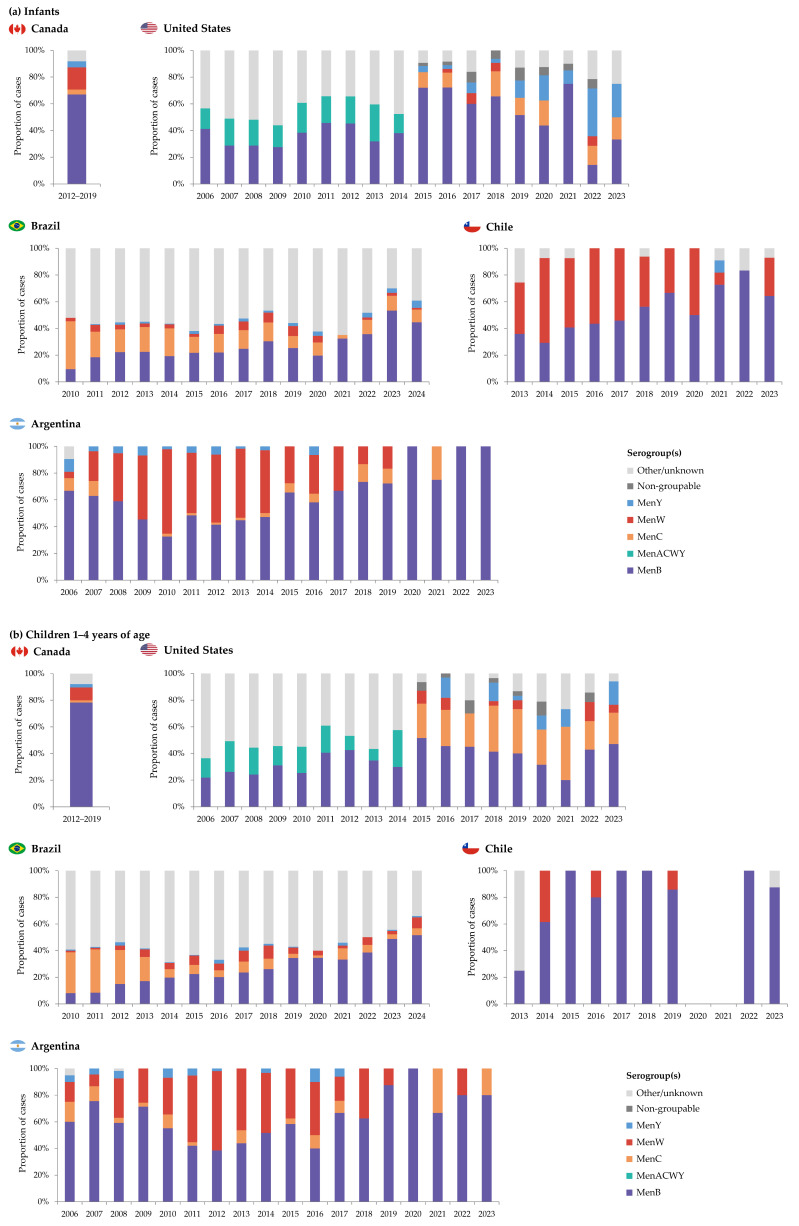
Distribution of IMD cases by serogroup in Canada, the United States, Brazil, Chile, and Argentina, by year, in infants (**a**) and children 1–4 years of age (**b**). IMD, invasive meningococcal disease. Note: Data publicly available for each country are presented. The serogroup category “Other/unknown” refers to serogroups other than those listed in the figure legend and instances where the IMD-causing serogroup was not determined. For Canada, serogroup distribution was only available for the 2012–2019 period, overall. For Chile, no IMD cases were listed for children 1–4 years of age in 2020 and 2021 (panel (**b**)). For Argentina, the data is for children 1–5 years of age for 2006–2008 (panel (**b**)).

MenB was the predominant serogroup [11] (Figure 3). From 2012 through 2019, the overall mean annual age-adjusted IMD incidence was 0.34/100,000 population. The highest incidence rates were observed among infants (3.56/100,000; ranging from 1.56 to 4.96 cases/100,000 population across years) and children 1–4 years of age (0.93/100,000, ranging from 0.51 to 1.18 cases/100,000 population) [13]. The same trend in distribution by age groups continued during the COVID-19 pandemic from 2020 to 2022; however, incidence rates were lower compared to the previous 3 years in infants (2.16, 1.94, and 1.63 cases/100,000 population in 2020, 2021, and 2022, respectively), but not in children 1–4 years of age (0.65, 0.39, and 0.60 cases/100,000 population) [21]. During 2012–2022, MenB was the most prevalent serogroup across all age groups, including for infants and children 1–4 years of age, with estimated incidence rates of 2.03 and 0.59/100,000 population, respectively [21].

An overall CFR of 13.8% was reported for the 2012–2019 period. CFRs varied by age group, with the highest CFR (19.0%) reported among infants [13].

IMD epidemiology varies by province/territory, prompting a recommendation from the National Immunization Technical Advisory Group (National Advisory Committee on Immunization [NACI]) for tailored regional decision-making on vaccination targeting the groups at the highest risk of disease [21]. Currently, vaccination against MenC is routinely recommended at 12 months of age; however, vaccination may begin as early as 2 months of age, depending on the provincial/territorial schedule and the MenC-IMD incidence in the respective areas [18] (Table 1). MenB vaccination may be considered on an individual basis in healthy infants and children 2–23 months of age [18], and MenB and/or MenACWY vaccination is also recommended for certain at-risk groups (Table 2). NACI advised that jurisdictions experiencing a higher incidence in specific populations might consider the implementation of targeted programs. These programs could provide MenB and/or MenACWY vaccines to age groups with the highest incidence of IMD, as well as to populations at higher risk of exposure (such as students residing in congregate settings or children and adolescents living in regions where hypervirulent clones are circulating) [21].

The unpredictability of IMD across Canada and the need for tailored vaccination programs are further illustrated by outbreaks occurring in various regions. Periodic MenC outbreaks continued to occur until 2006, mainly in unvaccinated individuals [27,28]. During 1999–2001, a large MenC outbreak in Alberta was successfully controlled with a mass vaccination campaign whereby a MenACWY vaccine was administered to individuals 2–24 years of age [29]. MenB was the cause of a large outbreak in Quebec during 2006–2013 and a university-based outbreak in Nova Scotia in 2015 [27]. In the Quebec’s Saguenay-Lac-Saint-Jean region, an immunization campaign targeting individuals aged between 2 months and 20 years was carried out starting in May 2014, using the four-component protein-based MenB vaccine (4CMenB). The MenB-IMD incidence in the region subsequently decreased by 92% from July 2014 to December 2016 compared to July 2006–June 2014, and no cases were reported in the age group targeted for vaccination [30]. An 87% decrease in MenB-IMD risk was estimated in the region over four years after the launch of the vaccination campaign (July 2014–June 2018) [31]. More recently, an increase in MenY incidence has been observed, beginning in 2017 and continuing to increase after the COVID-19 pandemic in Quebec [32]. This post-pandemic increase in IMD incidence rates, most probably caused by the lifting of preventive measures, was noted across Canada and accompanied by the shift in meningococcal serogroup prevalence. A series of smaller and largely MenB-associated outbreaks also occurred in post-secondary education settings in Atlantic Canada, sometimes leading to fatalities [33]. This resulted in the implementation of adolescent MenB vaccination programs in the provinces of Prince Edward Island and Nova Scotia [21]. In Manitoba, an increase in IMD cases caused mainly by MenW, was observed across all age groups since December 2023 and prompted the introduction of infant MenACWY vaccination as of March 2024. Thus, in this province, the MenC dose at 12 months was replaced by MenACWY vaccination, and catch-up programs were implemented for children born between 1 January 2020 and 28 February 2023 [34].

### 3.2. United States

In the US, IMD is a notifiable disease and is tracked using multiple surveillance systems, including the National Notifiable Diseases Surveillance System (NNDSS), Active Bacterial Core surveillance (ABCs; a network of laboratories in 10 areas of the country), and an enhanced surveillance system (since 2015) [35].

From 2006 to 2021, the overall incidence of IMD has decreased from 0.40 to 0.06 cases/100,000 population. However, an increase in IMD incidence has been subsequently observed, with 0.09 cases/100,000 reported for 2022 and 0.13 cases/100,000 population for 2023, largely due to an increased number of MenY cases [35]. The highest IMD incidence was observed in infants. In this age group, incidence rates peaked at 3.76 cases/100,000 population in 2008, gradually declining to 0.33/100,000 in 2023, although in 2021 the incidence was 0.56/100,000 population [36]. Lower incidence rates were observed in children 1–4 years of age during the same period, varying between 2.96 cases/100,000 population in 2008 to 0.09/100,000 population in 2022, and increasing again to 0.11/100,000 population in 2023 (Figure 2). MenB was the predominant serogroup in both age groups in most years [35] (Figure 3). In 2023, MenB was the predominant serogroup among infants (0.11/100,000 population) and children 1–4 years of age (0.05/100,000 population), with higher prevalence than in the overall population (0.02/100,000 population) [37]. Two fatalities occurred in infants in 2023 (CFR of 16.7% compared to 10.5% in the overall population), and one death occurred in the 1–4 years age group (CFR of 5.9%) [37].

Several IMD outbreaks have been reported in the US, including college/university outbreaks caused by MenB [38,39,40]. Since 2022, reported IMD outbreaks included a statewide MenY outbreak in Virginia, MenC and MenY outbreaks in people experiencing homelessness in New York and Colorado, MenW outbreaks in Iowa and in people traveling to Saudi Arabia, and a MenB outbreak in the Ohio Amish Community, affecting children 1–10 years of age [41]. In 2024, the Centers for Disease Control and Prevention issued an alert about the increase in MenY-IMD cases, mainly due to strain ST-1466 (March 2024) [42].

In the US, MenACWY vaccination is recommended for individuals at increased risk for IMD from 2 months of age (Table 2) [43]. Currently, no MenB vaccines are approved for use among children < 10 years old in the US [40].

### 3.3. Brazil

In Brazil, IMD is a notifiable disease, and data are publicly available since 2010 from the Notifiable Diseases Information System (SINAN) [19].

From 2010 (when MenC vaccination was introduced in the NIP [14]) to 2021, the overall incidence of IMD declined from 1.54/100,000 population to 0.12/100,000 population; the decline was more rapid in the first 4 years after MenC vaccine introduction, and another steep drop in incidence was observed from 2019 (0.51/100,000 population) to 2020 (0.17/100,000 population). As of 2022, IMD incidence increased again, to 0.39/100,000 population in 2024 [19].

IMD incidence rates were the highest among infants. In this group, incidence rates decreased sharply from 2010 (13.60/100,000 population) to 2012 (7.95/100,000 population) and then more steadily between 2013 and 2018 (from 7.65 to 4.59/100,000 population). IMD incidence was considerably lower during the COVID-19 pandemic (2.23 and 1.38/100,000 population in 2020 and 2021, respectively) compared to 2019 (Figure 2). Overall, as of 2012, MenB has been the most predominant serogroup in infants; however, for a substantial proportion of IMD cases (30.0–64.9%), the serogroup was not or could not be determined (Figure 3) [19]. In children 1–4 years of age, IMD incidence rates rapidly declined from 2010 (5.59/100,000 population) to 2016 (1.20/100,000), and then decreased steadily, with some variation, to 0.42 cases/100,000 population in 2021. Incidences in 2022–2024 were lower than pre-pandemic values (Figure 2). MenC was the predominant serogroup in this age group until 2013, and MenB has been predominant since 2014; however, for 34.0–68.8% of cases between 2010 and 2024, the serogroup was not or could not be determined (Figure 3) [19]. From 2020 to 2024, CFRs varied between 21.1% and 25.4% in the overall population, between 18.9% and 34.4% for infants, and 12.5% and 34.8% for children 1–4 years of age [19].

Several outbreaks, mostly due to MenC, were reported in Brazil between 2000 and 2010 [27]. In recent years, an outbreak due to MenC was reported in 2022 (Sao Paolo) [44] and MenB in 2023 (Alagoas) [45]. The incidence of IMD in 2024 was higher than in the previous years, which prompted some states to issue alerts on the importance of meningococcal vaccination [46].

In Brazil, MenC vaccination is routinely recommended for healthy children at 3, 5, and 12–15 months of age (Table 1), while MenACWY is recommended for individuals in high-risk groups at 2 and 6–9 months of age (Table 2) [24].

### 3.4. Chile

In Chile, IMD is a mandatory notifiable disease since 1976, and national data are collected for the Epidemiology Department at the Ministry of Health through the Institute of Public Health, the latter being responsible for the confirmation of meningococcal disease and surveillance of antimicrobial resistance [47,48].

From 2006 to 2010, the overall incidence of IMD peaked at 0.51 cases/100,000 population in 2007 and then declined to 0.33/100,000 population in 2010. Children < 5 years of age experienced the highest incidence rates [48]. Starting in 2011, the overall incidence of IMD increased again to 0.78/100,000 population in 2014 and then decreased to 0.36/100,000 population by 2019. During 2020–2022, lower IMD incidences were reported (0.03–0.15/100,000 population), but in 2023, the incidence was close to pre-pandemic levels (0.31/100,000 population) [47].

Information on age-specific incidence rates is publicly available from 2012. Incidence rates were considerably higher among infants but decreased from 2014 (16.63/100,000 population) to 2019 (6.52/100,000 population). During the COVID-19 pandemic, the incidence was 0.85/100,000 population in 2020 and increased to 4.63/100,000 population in 2021; a lower incidence was observed in 2023 (0.42/100,000 population) (Figure 2). In this age group, MenW was the predominant serogroup until 2017; MenB has accounted for 50.0–83.3% of yearly IMD cases ever since (Figure 3) [47]. In children aged 1–4 years, IMD incidence steadily decreased from 2014 to 2017 (from 1.31 to 0.30/100,000 population), increased until 2019 (0.72/100,000 population) and was 0.11/100,000 population in 2022 and 2023. No IMD cases were reported in this age group in 2020 and 2021 (Figure 2). Throughout 2013–2023, MenB was the serogroup causing most IMD cases in children 1–4 years of age (Figure 3) [47]. A median CFR of 19.9% was reported during the period 2010–2021 in the overall population, peaking in 2017 at 30.0% [14].

Outbreaks of IMD occurred periodically in Chile, including a large outbreak due to MenB in 1993 and one due to MenC in 2000 [49]. In 2012, MenW was the predominant serogroup, and the cause of an outbreak that affected mainly children < 5 years of age and adults ≥ 60 years of age. The outbreak was successfully controlled by introducing a MenACWY catch-up vaccination campaign among children 9 months to 5 years of age [15]. In June 2024, the Chilean Health Ministry issued an epidemiological alert regarding an unexpectedly high number of IMD cases occurring during the month of May and accumulated number of cases in the first 5 months of the year. Most of the 20 accumulated cases were due to MenB (55%) and MenW (40%), and five (25%) were in children < 5 years of age. Among these children, two were vaccinated with a meningococcal vaccine against serogroups other than the serogroups with which they were infected [50].

Single-dose MenACWY vaccination for 12-month-olds was introduced in the Chilean NIP in 2014 [15], prompted by a MenW outbreak that greatly impacted children < 5 years of age [51]. Subsequently, IMD incidence rapidly decreased, especially in young children. The median uptake rate of MenACWY vaccination was 95.6% during 2014–2021 [14]. In 2023, Chile became the first country in the Americas to have both MenACWY and MenB vaccination in the NIP. MenB infant vaccination was recommended as a three-dose schedule at 2 and 4 months of age with a booster dose at 18 months of age [52].

### 3.5. Argentina

In Argentina, IMD surveillance includes clinical and laboratory data. Notification of cases is performed through the National Surveillance System (SNVS) online platform, which the laboratories can access and complement the available information [12].

Until 2015, the overall incidence of IMD in Argentina varied between 0.4 and 0.7/100,000 population. A decreasing trend was observed since 2015 and reached an all-time low during the COVID-19 pandemic, with 20 cases (incidence of 0.05/100,000 population) notified country-wide in 2021. However, the incidence increased in 2022 (0.12/100,000) (Figure 2).

IMD incidence was highest among infants, with 14.2 cases/100,000 population in 2012. Incidence rates declined from ~8.0 to ~2.9 cases/100,000 population between 2015 and 2019 and continued to decrease through 2021. The incidence increased to 1.6/100,000 population in 2022 [20]. In infants, the predominant serogroup from 2009 to 2014 was MenW, and since 2015 it has been MenB, causing 58.1–100% of cases (Figure 3) [7,12,53,54,55,56,57,58,59,60,61,62,63,64,65].

IMD incidence was lower in children 1–4 years of age (Figure 2). Between 2015 and 2019, incidence rates varied from ~0.7 to 2.3 cases/100,000 population, peaking in 2017. In 2022, the IMD incidence in this age group was ~0.5/100,000 population [20]. Overall, MenB was the predominant serogroup between 2014 and 2023 [12,53,54,55,56,57,58,59,60,61,62,63,64,65] (Figure 3).

Between 2018 and 2022, CFRs varied from 3.3% to 15.6% in the overall population [20].

Official data on recent IMD outbreaks in Argentina are not available, but increases in MenW cases were reported between 2006 and 2008 [66].

MenACWY vaccination was introduced in Argentina in 2017, as three doses administered to children at 3, 5, and 15 months of age (Table 1), and as a single dose administered to children 11 years of age. Coverage for the first, second, and third dose in young children gradually increased between 2017 and 2022 and ranged between 75 and 85%, 56 and 80%, and 45 and 78%, respectively [20]. MenB vaccination is also recommended for high-risk groups [26] (Table 2).

### 3.6. Other Countries

#### 3.6.1. Cuba

In Cuba, IMD is a mandatory notifiable disease, and data are collected in a nationwide surveillance system [67]. Massive immunization campaigns against IMD were conducted in 1989–1990 with a MenBC vaccine, based on outer membrane proteins from MenB and containing MenC polysaccharide. These campaigns targeted children and adults from 3 months to 24 years of age and led to a steep decrease in IMD incidence, further accentuated after the introduction of MenBC vaccine in the NIP in 1991, given at 3 and 5 months of age [68].

Since 2008, the overall incidence of IMD has been maintained at <0.1/100,000 population [68]. In children ≤ 6 years of age, it is estimated that the IMD incidence decreased by 95% (93–98%) in the post-vaccination era compared to pre-vaccination [68].

In 2018, only one case of MenC-associated IMD was reported in children < 5 years of age [65]. In 2022 and 2023, the reported IMD incidence was 0.05/100,000 population, a slight increase from 0.02/100,000 population in 2020 [69].

#### 3.6.2. Costa Rica

In Costa Rica, IMD cases are reported to the Health Ministry. The country was also included in the SIREVA project from 1993 and is part of SIREVA II [70].

From 2006 to 2015, the overall IMD incidence was <1.0/100,000 population, with MenB causing 65% of cases, followed by MenY (22.5%) and MenC (7.5%). A MenB outbreak was reported in Heredia in 2006. MenB was also the most prevalent serogroup in children < 5 years of age [71].

Between 2006 and 2018, MenB was responsible for nearly all cases reported among infants (13/15 cases; 86.6%); with one (6.7%) MenY case in 2007 and one (6.7%) non-groupable case in 2014. In children 1–4 years of age, only eight cases were reported in this period: three (37.5%) were caused by MenB, two (25.0%) by MenY, and three (37.5%) non-groupable cases. No data are reported for Costa Rica in 2015 [53,54,55,56,57,58,59,60,61,62,63,64,65].

In 2021, the overall IMD incidence was <0.5/100,000 population, based on data from the Costa Rican Institute of Research and Teaching in Nutrition and Health (INCIENSA). MenB was the most predominant serogroup overall, although increases in MenW- (since 2018) and MenY-IMD were also observed [72]. Based on World Health Organization (WHO) data, IMD incidence was 0.02/100,000 population in 2020 and 0.04/100,000 population in 2023, but age-specific incidences are not available [69].

#### 3.6.3. Honduras

Available data on IMD incidence are scarce for Honduras, although the country is included in the SIREVA II network, and surveillance of *N. meningitidis* began in 2000 [73].

Between 2006 and 2018, only one non-groupable case of IMD was reported in children < 5 years (in an infant) [53,54,55,56,57,58,59,60,61,62,63,64,65]; after 2018, based on WHO data, no cases were reported through 2023 and the overall incidence of IMD was 0.04/100,000 population [69].

#### 3.6.4. Panama

In Panama, reporting of IMD cases has been mandatory as of 2014 and is centralized by the Health Ministry and the Gorgas Institute for Health Studies [74].

The overall incidence of IMD was 1/100,000 people in 2008 and has further declined since, with low incidences reported between 2009 and 2021. All cases reported between 2013 and 2021 were caused by MenB [72]. Nine IMD cases were reported in children ≤ 5 years of age in 2008, six of which occurred in infants and were due to MenB (two) and MenC (four cases). The three cases in children 1–5 years were also caused by MenB (one) and MenC (two cases) [55]. In 2009, four MenC cases were reported in children 1–4 years of age [56]. MenC continued to be the predominant cause of IMD in children < 5 years of age in 2010 [57] and in 2011 [58]. Between 2012 and 2018, only one case of IMD (caused by MenB in an infant in 2017) was reported among children aged <5 years [58,59,60,61,62,63,64,65]. IMD incidence remains low, with only one case reported in both 2023 and 2024 so far [74].

#### 3.6.5. Colombia

In Colombia, IMD cases are collected via the national surveillance system, which has been mandatory since 1985 [75].

The reported overall incidence of IMD was 0.33–0.45/100,000 population during 2005–2011 [75] and 0.04–0.18/100,000 population during 2015–2021 [10]. However, for the 2005–2011 period, only 46% of cases were confirmed [75]. Throughout both periods, the highest IMD incidence was in infants (5.4–6.9/100,000 population during 2007–2011 and 0.52–1.47/100,000 population during 2015–2021), followed by children aged 1–4 years of age (0.5–0.9/100,000 population during 2007–2011 and 0.03–0.47/100,000 population during 2015–2021) [10,75].

During 2015–2021, the highest incidence of fatal IMD was among infants, with a mean of 0.30/100,000 population, although this varied by year, from 0.00 to 0.65/100,000. Children aged 1–4 years experienced the second highest mortality rate, with a mean of 0.07/100,000 compared to 0.01–0.05/100,000 population in the older age groups [10].

Overall, MenB was the predominant serogroup causing IMD in Colombia from 1988 to 2005, in 2007, and from 2009 to 2014; MenY was predominant in 2006 and 2008, while MenC became the dominant serogroup from 2015 to 2021 [10]. During 2015–2020, MenC accounted for 40.0% and 56.3% of IMD cases in infants and children 1–4 years of age, respectively. The second most predominant serogroup was MenB: 18.6% in <1-year-olds and 27.1% in 1–4-year-olds, although 34.3% of serogroups causing IMD in infants and 12.5% in children 1–4 years of age were non-typable [10]. Several outbreaks were reported in Colombia, mainly due to MenB and MenC, followed by MenY [10].

#### 3.6.6. Uruguay

IMD is a mandatory notifiable disease in Uruguay, but publicly available data are scarce. During 2010–2021, the median IMD incidence was 0.47/100,000 population, and a steep increase was observed after 2016, with an incidence reaching 0.88/100,000 population in 2019. A 72.6% decrease was observed in 2020 (during the COVID-19 pandemic) compared to the median in the period 2010–2019. The incidence was highest in infants and children 1–4 years of age, and MenB was the most predominant serogroup [14]. More recently, the number of IMD cases increased in 2024, particularly in children < 5 years of age [76]. Up to week 38 of 2024, 35 IMD cases were reported, compared to 20–30 cases that were previously reported yearly, with a CFR of 23% [76,77]. Following increased IMD incidence, the Uruguayan Vaccination Advisory Committee recommended the introduction of meningococcal vaccination in the NIP, with MenB and MenACWY vaccination to be administered in children < 2 years of age. MenACWY vaccination was also recommended for adolescents 11 years of age [77].

## 4. Discussion

Almost a decade ago, the WHO launched a call to action to establish a global strategy aimed at eliminating meningitis as a public health concern. This resulted in the development of a global road map to defeat meningitis by 2030. Key objectives of this road map included eliminating bacterial meningitis epidemics, reducing the incidence of vaccine-preventable bacterial meningitis by 50% and reducing related deaths by 70%, as well as decreasing disability and improving the quality of life for individuals affected by meningitis [78]. The control of epidemics and prevention of meningitis can be achieved by improving prevention strategies and attaining high vaccine coverage. For meningococcal disease, one of the milestones set by the WHO for 2024 was to introduce or maintain locally relevant vaccination strategies, based on the regional epidemiology [78].

In the Americas, as with many infectious diseases, children < 5 years of age bear the highest incidence and burden of IMD. Moreover, the prevalence of MenB is disproportionally higher among both infants and children 1–4 years of age, and recurrent MenB-IMD outbreaks and high CFRs are observed in these age groups. However, despite overwhelming evidence of a medical need for children < 5 years of age and the availability of effective vaccines [79], only five countries in the Americas have implemented programmatic vaccination against IMD for this age group. Additionally, most of the vaccination programs target IMD caused by MenC or MenACWY and not MenB which causes most of the disease, except in Cuba where MenBC vaccination has been available in the NIP since 1991. Chile introduced infant MenB vaccination programs into its NIP in 2023.

In this respect, countries in the Americas lag behind Europe, where at least 14 countries have introduced MenB vaccination for infants in their NIP [80,81], since the first NIP was implemented in the United Kingdom (UK) in 2015 [82]. In most of the NIPs, infant MenB vaccination is administered as a two-dose primary series as of 2 months of age, with a booster dose at 12–15 months. Several European countries recommend MenC/MenACWY vaccination in children < 5 years of age, although most of these countries also recommend MenB vaccination. Often, MenACWY vaccination recommendations have replaced previous MenC vaccination programs, ensuring ongoing protection against MenC while also addressing increasing incidence of IMD caused by other serogroups [83]. There is ample evidence from the European region demonstrating that vaccination in young children, tailored to local epidemiology, can help reduce the incidence of IMD caused by all Men serogroups [8,80,83]. While several countries in the Americas have successfully used meningococcal vaccination for outbreak control, only vaccination recommendations in Chile align with European strategies targeting this age group. Conversely, Canada and Brazil continue to focus on MenC-IMD, despite the increasing prevalence of other serogroups.

During 2020–2022, COVID-19 lockdown measures and other mitigation strategies led to significantly lower global incidences of IMD [84]. Similar to trends observed for other pathogens upon lifting of restrictions, several post-pandemic IMD outbreaks have been reported globally, including in countries in the Americas [85]. In Europe, post-pandemic IMD incidence rates were sometimes higher than pre-pandemic ones, especially among populations not targeted by existing meningococcal vaccination programs [86,87]. For the five countries in the Americas that were the focus of our review, although increases were observed since 2022, IMD incidence rates have yet to return to pre-pandemic levels for both infants and children 1–4 years of age. During the COVID-19 pandemic, MenB was the predominant serogroup causing IMD in many countries in the Americas, where vaccination strategies for MenB-IMD in young children are largely lacking. The absence of vaccination strategies may have represented a missed opportunity to sustain the low IMD incidence observed during the pandemic, thereby achieving greater control of the disease, particularly among young children who are at higher risk of IMD.

Because 4CMenB was not shown to have an effect on meningococcal carriage and its effect is believed to rely on direct protection [88,89], achieving high vaccine uptake is essential. In Italy, 4CMenB was introduced in the NIP in 2017, with varying ages at administration across regions. Despite a national vaccination coverage of only 38.6% in 2017 [90], the number of MenB cases decreased by 91% in Tuscany and by 80% in Veneto among vaccinated children compared to the pre-vaccine era (although the estimated vaccine coverages in these regions were >80%) [91]. These data indicate that even suboptimal 4CMenB coverage can lead to significant reductions in MenB-IMD incidence. However, the ultimate goal should be implementing a comprehensive vaccination program with high coverage, to maximize protective benefits and eventually eliminate IMD in young children.

The known continuous variation in IMD epidemiology prompts for strategies to protect against all five most common Men serogroups. Optimal vaccination uptake can be facilitated by co-administration of meningococcal vaccines with other routine immunizations or among themselves. Previous evidence shows that MenB and MenACWY vaccines can be administered with other pediatric vaccines without a clinically relevant effect on immune responses or safety/reactogenicity [92,93,94]. Furthermore, a combined vaccination strategy, including direct protection against MenB of infants and toddlers and catch-up or additional MenB and/or MenACWY vaccination in adolescence, could improve control and prevention of IMD [95,96]. Such a complementary vaccination strategy would mitigate the lack of effect of 4CMenB on MenB carriage and herd immunity. A dynamic transmission model adapted for Argentina indicated that incorporating infant 4CMenB in the NIP could substantially reduce the burden of IMD. Additionally, maintaining adolescent MenACWY vaccination could achieve near-elimination of MenACWY-IMD within 30 years [97].

Despite the availability of meningococcal vaccines, barriers to mass vaccination strategies that would afford equitable access for the entire population still exist. Introduction of MenB vaccines in NIPs or publicly funded regional programs has proven more difficult than MenC and MenACWY vaccination due to their higher cost. Countries such as the UK and France initially deferred the implementation of MenB vaccines in their NIPs, citing low cost-effectiveness as a reason. However, they subsequently reconsidered this decision, due to the severity of the disease, the potential long-lasting effects of IMD (as well as their economic burden), loss of quality of life to the patients and their families, and the need to make the vaccine accessible to low-income populations that may be disproportionately impacted by the disease [98]. In England, 5 years after the introduction of the MenB program, a cost-effectiveness analysis that more comprehensively accounted for the burden of MenB-IMD showed that 4CMenB infant vaccination can be cost-effective [99]. Drawing from the European experience, more countries in the Americas are now well-positioned to implement comprehensive meningococcal vaccination programs, particularly in children < 5 years of age that are most impacted by IMD.

Our study is not without limitations. The five countries discussed here might not be entirely representative of the current epidemiologic context of the Americas. Moreover, epidemiology data were not available in a consistent manner for all these countries. Data were derived from different, country-specific sources and starting from different dates. Another main challenge was the lack of consistent availability of more granular data, as data was often reported in aggregate form. As a result, information regarding serogroup-specific cases, age demographics, and annual trends was frequently unavailable. Surveillance systems differ from one country to another, which may impact a direct comparison. Additionally, there is an inherent lag between collecting, reporting, and publishing surveillance data, which might affect our interpretation of the more recent data (published in 2024–2025).

## 5. Conclusions

Vaccination programs targeted at age groups with the highest incidence of vaccine-preventable serogroups, in particular MenB, could help reduce the IMD burden in young children in the Americas. Comprehensive NIPs and national recommendations could facilitate equitable access to protection against IMD, aligned to the WHO roadmap to defeat meningitis by 2030. The European experience shows that national vaccination programs against meningococcus can be successful.

## Figures and Tables

**Table 1:** National/publicly funded routine recommended vaccinations against IMD in infants and/or young children in Canada, Brazil, Chile, and Argentina.

Country	Vaccine	Schedule	Year of Implementation
Canada ^1^	MenC	Single dose at 12 months	2002
	MenACWY (Manitoba only)	Single dose at 12 months ^2^	2024
Brazil	MenC	3 doses at 3, 5, and 12–15 months	2010
Chile	MenACWY	Single dose at 12 months	2014
	MenB	3 doses at 2, 4, and 18 ^3^ months	2023
Argentina	MenACWY	3 doses at 3, 5, and 15 months	2017

^1^ MenB vaccination may be considered on an individual basis, depending on individual preferences, regional MenB epidemiology and strain susceptibility; ^2^ Replacing MenC vaccination previously recommended at the same age; ^3^ The booster dose was introduced in November 2024. IMD, invasive meningococcal disease.

**Table 2:** National high-risk group recommended vaccinations against IMD in infants and/or young children in Canada, the United States, Brazil, Chile, and Argentina.

		Canada		United States	Brazil	Chile	Argentina	
		MenACWY	MenB	MenACWY	MenACWY	MenACWY	MenACWY	MenB
Underlying condition								
	Anatomic or functional asplenia ^1^	✓	✓	✓		✓	✓	✓
	Persistent complement component deficiency and/or complement inhibitor	✓	✓	✓		✓	✓	✓
	HIV infection	✓	✓	✓		✓	✓	✓
	Combined T and B cell immunodeficiencies or primary antibody deficiencies	✓	✓			✓		
	Cochlear implant recipients and/or CSF leak	✓ ^2^	✓ ^2^			✓		
	BMT and SOT patients					✓		
	Preterm babies with underlying conditions					✓		
	Patients with nocturnal paroxysmal hemoglobinuria				✓			
Vaccination schedule		2–11 MOA: 2 or 3 doses (given 8 w apart, with another dose between 12 and 23 MOA, given at ≥8 w from previous dose) 1–4 YOA: 2 doses given ≥8 w apart		2–4 doses given ≥8 w apart, depending on the vaccine and age at first vaccination+ booster 3 Y later and every 5 Y hereafter	2 doses at 2 MOA and 6–9 MOA	<1 YOA: 4 doses at 2, 4, 6, 12 MOA + booster 3–5 Y later ≥1 YOA: 2 doses + booster 3–5 Y later 1 dose for children with cochlear implant	2–6 MOA: 3 doses + booster at 12–16 MOA 7–23 MOA: 1 dose + booster at 12–23 MOA, ≥2 M apart ≥2 YOA: 1 dose (+ booster for children 2–5 YOA at continuous increased IMD risk).	2 MOA: 3 doses + booster at 12–23 MOA 3–5 MOA: 2 doses + booster at 12–23 MOA 6–11 MOA: 2 doses + booster ≥2 M later 12–23 MOA: 2 doses + booster ≥12 M later ≥2 YOA: 2 doses
Reference		[18,22]		[23]	[24]	[25]	[26]	

BMT, bone marrow transplantation; CSF, cerebrospinal fluid; IMD, invasive meningococcal disease; M, months; MOA, months of age; w, weeks; SOT, solid organ transplantation; Y, year; YOA, year of age. ^1^ Including sickle cell disease; ^2^ In Ontario only. Note: ✓ indicates that vaccination against select meningococcal serogroups was introduced in a given country.

## Data Availability

Data presented in this study are included in the article and relevant references. Further inquiries can be directed to the corresponding author(s).

## Abbreviations

The following abbreviations are used in this manuscript:

CFR	Case fatality rates
IMD	Invasive meningococcal disease
IR	Incidence rate
NIP	National immunization programs
NNDSS	National Notifiable Diseases Surveillance System
Men	Meningococcal serogroup
UK	United Kingdom
US	United States
WHO	World Health Organization
